# Possible adverse events of imidazole antifungal drugs during treatment of vulvovaginal candidiasis: analysis of the FDA Adverse Event Reporting System

**DOI:** 10.1038/s41598-024-63315-1

**Published:** 2024-06-24

**Authors:** Tianyu Zhou, Chongze Chen, Xiaowei Chen, Bin Wang, Feng Sun, Wanfang Li, Dong Liu, Hongtao Jin

**Affiliations:** 1 Department of Pharmacy, Shaanxi University of Chinese Medicine, Xianyang, China; 2 Key Laboratory of Pharmacodynamics and Material Basis of Chinese Medicine of Shaanxi Administration of Traditional Chinese Medicine, Xianyang, China; 3 Engineering Research Center of Brain Health Industry of Chinese Medicine, Universities of Shaanxi Province, Xianyang, China; 4 New Drug Safety Evaluation Center, Institute of Materia Medica, Chinese Academy of Medical Sciences and Peking Union Medical College, Beijing, China; 5 Department of Pharmacy, Fuzhou Changle People’s District Hospital, Fuzhou, Fujian China; 6 Department of Epidemiology and Biostatistics, School of Public Health, Peking University, Beijing, China; 7 NMPA Key Laboratory for Safety Research and Evaluation of Innovative Drug, Beijing, China; 8 Beijing Union Genius Pharmaceutical Technology Development Co. Ltd, Beijing, China; 9 Center for Drug Evaluation, NMPA, Beijing, China

**Keywords:** Diseases, Medical research, Risk factors

## Abstract

Azole antifungal drugs are commonly used to treat vulvovaginal candidiasis (VVC). The nephrotoxicity and developmental toxicity of azole drugs have not been systematically analyzed in the real world. We used the FDA Adverse Event Reporting System (FAERS) to investigate the adverse events (AEs) associated with imidazole therapy for VVC. FAERS data (from quarter 1 2004 to quarter 3 2022) were retrieved using OpenVigil 2.1, and AEs were retrieved and standardized according to the Medical Dictionary for Regulatory Activities (MedDRA). In the top 10 System Organ Class (SOC), all four drugs have been found to have kidney and urinary system diseases and pregnancy. We found significant signals, including clotrimazole [bladder transitional cell carcinoma, (report odds ratio, ROR = 291.66)], [fetal death, (ROR = 10.28)], ketoconazole[nephrogenic anemia (ROR = 22.1)], [premature rupture of membranes (ROR = 22.91 46.45, 11, 3)], Miconazole[hematuria (ROR = 19.03)], [neonatal sepsis (ROR = 123.71)], [spontaneous abortion (ROR = 5.98)], Econazole [acute kidney injury (ROR = 4.41)], [spontaneous abortion (ROR = 19.62)]. We also discovered new adverse reactions that were not reported. Therefore, when using imidazole drugs for treatment, it is necessary to closely monitor the patient's renal function, pay attention to the developmental toxicity of the fetus during pregnancy, and be aware of potential adverse reactions that may occur.

## Introduction

Vulvovaginal candidiasis (VVC) is a common mucosal fungal infection among women of childbearing age, and most women experience one or two episodes in their lifetime^1–3^. A case of fetal right kidney dysplasia after fluconazole treatment of VVC during pregnancy was found in the database of Fujian Provincial Fuzhou Changle People’s District Hospital, China. Ultrasound examination of the newborn infant showed polycystic kidney disease. With the widespread use of imidazole drugs, drug related adverse reactions (AEs) have also received attention. Pregnancy has been found to be an increasingly important factor in the incidence of VVC^4^. With the continuous improvement of medical and social conditions, the issue of perinatal medication has received increasing attention. A recent meta-analysis showed that the overall prevalence of VVC among pregnant women was 29.2% (CI 95%: 23.4–33.0)^5^.

The 2015 Guidelines for the Treatment of Sexually Transmitted Diseases, released by the Centers for Disease Control and Prevention (CDC) in the United States, recommend a 7-day dose of vaginal azole medication. At present, many imidazole drugs have been approved by the FDA, and the imidazole derivatives such as clotrimazole, econazole, ketoconazole and miconazole are approved for topical treatment of genital candidiasis^4,6^. In a microbial community experiment of VVC patients, it was found that imidazole drugs (econazole, clotrimazole, miconazole, and ketoconazole) were sensitive to isolated strains at lower concentrations (MIC ≤ 1 μg/ml), which means that these drugs are effective in 1 μg/ml or lower, the growth of the majority of tested fungal strains can be inhibited, while other drugs have no effect on bacterial activity at this concentration^7,8^. The most common AEs of imidazole drugs include skin itching and allergic reactions. Due to insufficient research on the safety of azole drugs in reproductive development, we cannot completely rule out the possibility that they may lead to unknown adverse reactions related to reproductive development. Therefore, we used the FDA Adverse Event Reporting System (FAERS) database for pharmacovigilance analysis to better understand the adverse effects and relevance of imidazole drugs^6,9^. FAERS is one of the largest AE databases designed to support the FDA’s post-marketing safety oversight program for approved drugs and biologics^10,11^. Data mining techniques, such as signal detection algorithms, are increasingly being used to explore medical databases and analyze vast amounts of accumulated data to identify potential associations between drugs and AEs that may evade detection in clinical trials^12^.

## Methods

### Data sources

The data for this study was retrieved from the publicly available FAERS database, which follows the International Conference on Harmonization (ICH E2B) guidelines for international safety reporting^13^. Due to the voluntary nature of the report, the reporter may not be able to provide complete patient information, medication use history, and details of adverse events. In addition, adverse reactions may be related to the underlying condition being treated, caused by taking other medications at the same time, or occur for other reasons. The information in these reports reflects only the reporter's observations and opinions. The subjective judgment and interpretation of the reporter may also have an impact on the accuracy of the data. Therefore, when using FAERS data for causal relationship assessment, it is necessary to carefully review and verify the data. Classification and Standardization of AEs in FAERS are according to the Medical Dictionary of Regulatory Activities (MedDRA)^14^. We used the open tool OpenVigil 2.1 (https://openvigil.sourceforge.net/) to query the FAERS database. OpenVigil 2.1 is a tool for extracting, cleaning, mining, and analyzing pharmacovigilance data in FAERS^15^. FAERS data from the first quarter of 2004 to the third quarter of 2022 are currently included. In the OpenVigil database, the generic names of the target drugs were clotrimazole, econazole, ketoconazole and miconazole, gender was restricted to female, and we searched for AEs related the four drugs.

### Data mining

Report odds ratio (ROR)^16^ and Proportion report ratio (PRR)^17^ were used for data detection; the larger the ROR and PRR values, the stronger the signal, indicating a stronger statistical relationship between the target drug and AEs^18^. ROR and PRR are both frequency methods, which can estimate relative risk and reduce bias due to the selection of control groups. They are characterized by simple calculation and good consistency of results. The Bayesian Confidence Propagation Neural Network^19^ adopts a neural network supervised learning method, using known adverse drug reactions as a machine learning training set, and the detected adverse reactions have a strong correlation^20^. We used the above three methods as conditions for screening imidazole effective signal generation (Supplementary Table S1).

### Informed consent

All of our authors have read the final manuscript and agree to submit it.

## Results

### Descriptive characteristics

In the FAERS database, each report is encoded using the preferred term (PT) in MedDRA^21^. A given PT can be assigned to one or more High Level Term, High Level Group Term, and System Organ Class (SOC) levels in MedDRA. We used MedDRA 25.1 to standardize the analysis of adverse drug event signals and analyze SOC and PT according to the case report in the database.

**Figure 1:**
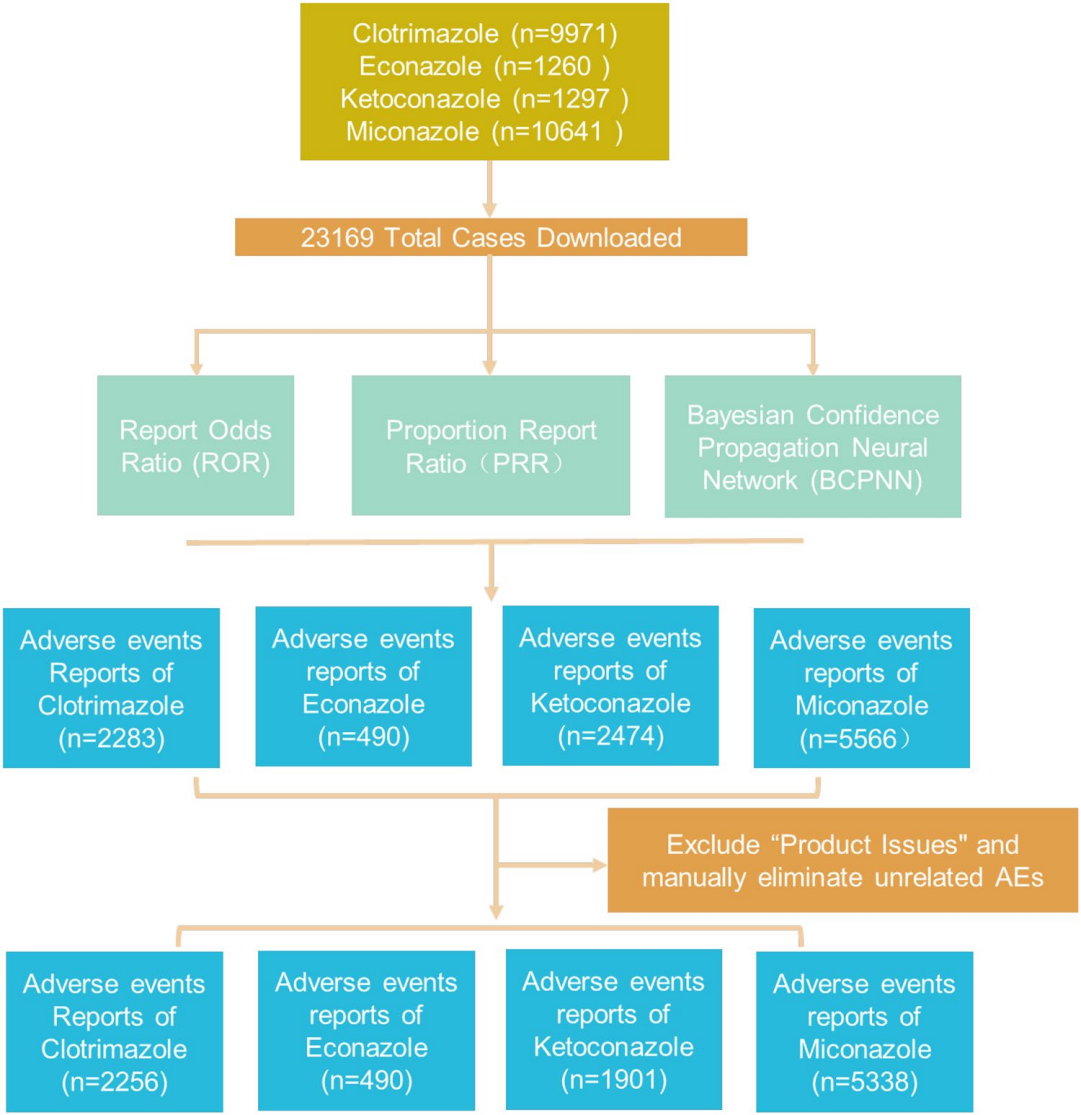
Identification process for post-marketing reports of suspected AEs related to clotrimazole, econazole, ketoconazole and miconazole, from the first quarter of 2004 to the third quarter of 2022.

We collected a total of 23, 169 AE reports. After excluding product problems and manual exclusion of unrelated AEs, the number of AEs reported with positive signals screened according to the inclusion criteria was clotrimazole 2256, econazole 490, ketoconazole 1901 and miconazole 5338. Clotrimazole accounted for 22.59%, econazole for 4.91%, ketoconazole for 19.04%, and miconazole for 53.46% (Fig. 1). The age distribution of patients with AEs was mainly in those aged ≥ 19 years: 19–39 years (2169 cases, 21.72%), 45–59 years (2171 cases, 21.74%), and ≥ 60 years (1948 cases, 19.51%). Severe AEs accounted for 31.92% (3187 cases) of the total AEs: Clotrimazole: 1407/2256 (62%); Miconazole: 740/5338 (13%); Ketoconazole: 675/1901 (35%); Econazole: 365/490 (74%), (Table 1).

**Table 1:** Summary of AE reports submitted in FAERS.

Baseline information	Total	Clotrimazole	Econazole	Ketoconazole	Miconazole
Number of AEs reports (n, %)					
Total	9985	2256 (22.59)	490 (4.91)	1901 (19.04)	5338 (53.46)
0–18	206 (2.06)	36 (1.60)	0 (0)	15 (0.79)	155 (2.90)
19–39	2169 (21.72)	392 (17.38)	50 (10.20)	288 (15.15)	1439 (26.96)
40–59	2171 (21.74)	661 (29.30)	28 (5.71)	639 (33.61)	843 (15.79)
≥ 60	1948 (19.51)	464 (20.57)	286 (58.37)	483 (25.41)	715 (13.39)
Abnormal or missing	3491 (34.96)	703 (31.16)	126 (25.71)	476 (25.04)	2186 (40.95)
Severity outcome (n, %)					
Total	3187 (31.92)	1407 (14.09)	365 (3.66)	675 (6.76)	740 (7.41)
Death	546 (5.47)	339 (3.04)	109 (1.09)	53 (0.53)	45 (0.45)
Disability	262 (2.62)	102 (1.02)	19 (0.19)	78 (0.78)	63 (0.63)
Hospitalization	1852 (18.55)	756 (7.57)	205 (2.05)	391 (3.92)	500 (5.01)
Life-threatening	527 (5.28)	210 (2.10)	32 (0.32)	153 (1.53)	132 (1.32)

### Drug safety signals

We screened developmental toxicity keywords as low level terms and obtained 312 developmental toxicity keywords (Supplementary Table S2). Renal and urinary disorders were mapped based on the classification in MedDRA. We discovered some unexpected important safety signals. Renal failure has been found as an AE for various drugs, and includes chronic kidney disease and acute kidney injury. We found that patients using clotrimazole were more likely to develop bladder transitional cell carcinoma [291.66 (613.67, 138.62)], and fetal death [10.28 (24.92, 4.24)] based on ROR signal strength. Patients using ketoconazole were more likely to develop nephrogenic anemia [22.1 (59.36, 8.23)] and premature rupture of membranes [22.91 (46.45, 11.3)]. Miconazole was more likely to cause hematuria [19.03 (30.48, 11.88)], neonatal sepsis [123.71 (274.76, 55.7)] and spontaneous abortion [5.98 (16.02, 2.24)]. Econazole was more likely to cause acute kidney injury [4.41 (9.97, 1.95)] and spontaneous abortion [19.62 (41.34, 9.31)] in clinical trials. These results indicate that these drugs can cause nephrotoxicity in clinical trials, and we should pay attention to related AEs (Table 2).

**Table 2:** Occurrence of drugs in different disorders.

Drug	Age	Case	PT name	ROR
Pregnancy, puerperium and perinatal conditions				
A	19–39	5	Foetal death	10.28 (24.92, 4.24)
B	19–39	9	Abortion spontaneous	19.62 (41.34, 9.31)
D	19–39	8	Premature rupture of membranes	22.91 (46.45, 11, 3)
C	0–18	6	Drug withdrawal syndrome neonatal	20.14 (45.92, 8.83)
C	0–18	7	Sepsis neonatal	123.71 (274.76, 55.7)
C	19–39	7	Premature baby	7.54 (15.93, 3.57)
C	19–39	13	Premature labour	4.96 (8.59, 2.86)
C	40–59	4	Abortion spontaneous	5.98 (16.02, 2.24)
Renal and urinary disorders				
A	0–18	3	Dysuria	28.46 (90.85, 8.92)
A	19–39	8	Pelvic pain	4.05 (8.18, 2.01)
A	19–39	4	Renal failure	4.51 (12.1, 1.68)
A	19–39	3	Urinary retention	7.46 (23.29, 2.39)
A	40–59	19	Acute kidney injury	10.45 (16.57, 6, 59)
A	40–59	22	Chronic kidney disease	19.96 (30.71, 12.97)
A	40–59	8	End stage renal disease	31.35 (63.37, 15.51)
A	40–59	5	Nephrogenic anaemia	55.42 (135.11, 22.73)
A	40–59	6	Pelvic pain	18.69 (41.96, 8.32)
A	40–59	3	Polyuria	19.46 (60.85, 6.22)
A	40–59	18	Renal failure	8.66 (13.9, 5.4)
A	40–59	4	Renal impairment	3.93 (10.52, 1.47)
A	40–59	4	Renal injury	6.63 (17.78, 2.47)
A	40–59	4	Renal tubular necrosis	21.92 (58.92, 8.15)
A	40–59	5	Tubulointerstitial nephritis	18.85 (45.67, 7.78)
A	≥ 60	8	Bladder transitional cell carcinoma	291.66 (613.67, 138.62)
A	≥ 60	3	Chromaturia	4.05 (12.6, 1.3)
A	≥ 60	18	Chronic kidney disease	11.63 (18.6, 7.27)
A	≥ 60	5	Dysuria	4.34 (10.47, 1.8)
A	≥ 60	4	End stage renal disease	10.57 (28.33, 3.95)
A	≥ 60	6	Hyperparathyroidism secondary	59.47 (134.37, 26.32)
A	≥ 60	8	Incontinence	19.25 (38.78, 9.56)
A	≥ 60	4	Nephrogenic anaemia	21.35 (57.33, 7.95)
A	≥ 60	10	Pollakiuria	7.01 (13.1, 3.75)
A	≥ 60	5	Renal injury	7.67 (18.51, 3.18)
A	≥ 60	5	Tubulointerstitial nephritis	8.54 (20.63, 3.54)
A	≥ 60	6	Urinary incontinence	5.11 (11.43, 2.29)
A	≥ 60	4	Urinary retention	4.49 (12.02, 1.68)
B	≥ 60	6	Acute kidney injury	4.41 (9.97, 1.95)
B	≥ 60	4	Renal failure	3.36 (9.06, 1.25)
B	≥ 60	6	Urinary tract infection	3.07 (6.94, 1.36)
D	19–39	5	Renal failure	6.88 (16.69, 2.84)
D	40–59	8	Acute kidney injury	4.1 (8.26, 2.04)
D	40–59	3	Chromaturia	5.95 (18.56, 1.91)
D	40–59	14	Chronic kidney disease	11.92 (20.32, 6.99)
D	40–59	3	Haematuria	7.07 (22.05, 2.27)
D	40–59	6	Pelvic pain	17.95 (40.29, 8)
D	40–59	11	Renal failure	4.99 (9.08, 2.74)
D	40–59	3	Renal injury	4.76 (14.85, 1.53)
D	≥ 60	17	Chronic kidney disease	11.36 (18.41, 7.01)
D	≥ 60	6	End stage renal disease	16.51 (37.01, 7.37)
D	≥ 60	4	Nephrogenic anaemia	22.1 (59.36, 8.23)
D	≥ 60	3	Renal injury	4.74 (14.76, 1.52)
C	0–18	3	Renal failure	4.92 (15.47, 1.56)
C	19–39	19	Dysuria	12.27 (19.42, 7.76)
C	40–59	4	Cystitis haemorrhagic	52.26 (141.78, 19.27)
C	40–59	9	Dysuria	8.78 (16.98, 4.54)
C	40–59	8	Haematuria	13.4 (26.98, 6.65)
C	≥ 60	4	Chronic kidney disease	2.88 (7.7, 1.08)
C	≥ 60	10	Dysuria	10.02 (18.75, 5.36)
C	≥ 60	18	Haematuria	19.03 (30.48, 11.88)
C	≥ 60	4	Pollakiuria	3.17 (8.47, 1.18)

A, clotrimazole; B, econazole; C, miconazole; D, ketoconazole.

### AE signal detection of drugs

**Figure 2:**
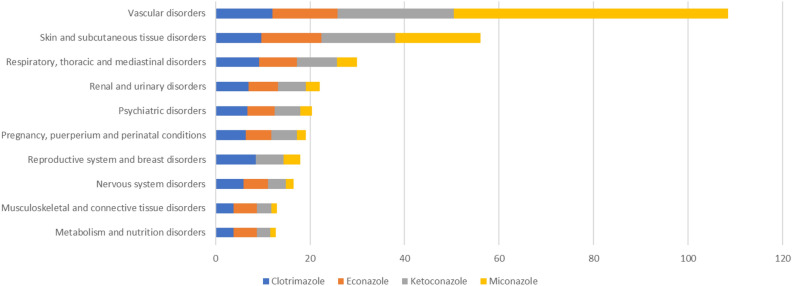
SOC distribution of AEs signal proportion.

In this study, we conducted signal detection on targeted drugs to identify specific clinical cases associated with AEs related to clotrimazole, econazole, miconazole and ketoconazole. We found 9985 AE signals, mainly involving 23 SOCs. The SOC signals of the four imidazole drugs mainly included vascular disorders; skin and subcutaneous tissue disorders; Respiratory, thoracic and mediastinal disorders; renal and urinary disorders; psychiatric disorders; pregnancy, puerperium and perinatal conditions; reproductive system and breast disorders; and so on. Among them, renal and urinary disorders; pregnancy, purperium and perinal conditions signals were found in the top ten SOC distributions, indicating that paying attention to related adverse reactions is a key issue for future research (Fig. 2).

### Relationship between main AE signals detection and SOCs

**Figure 3:**
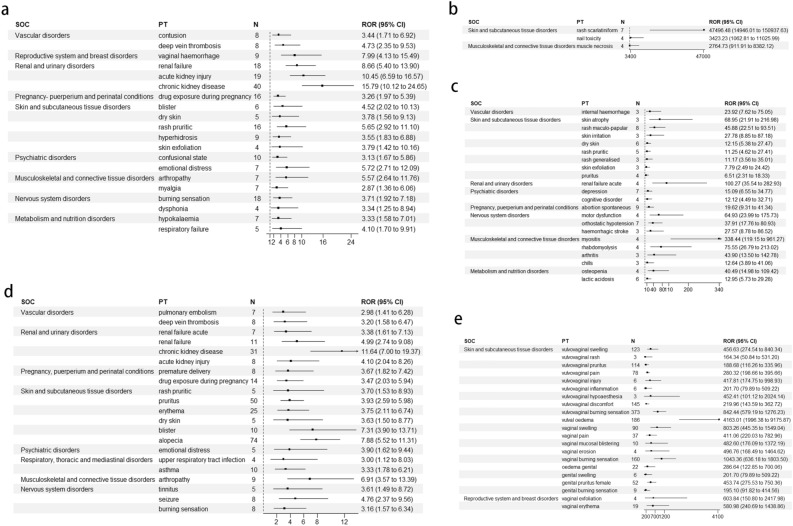
Main SOC and signal detection results of AE reports. (**a**) clotrimazole. (**b, c**) econazole, (**d**) ketoconazole, (**e**) miconazole.

According to the top ten SOC signals, AEs signals are detected according to PT, and the AEs of all detected signals are sorted by the top 50 in order of relevant intensity (lower limit of the 95% CI of the ROR estimates ). For clotrimazole, the strongest signal [ROR = 15.79 (10.12, 24.65)] and highest frequency (40 cases) were for chronic kidney disease, followed by acute kidney injury [ROR = 10.45 (6.59, 16.57)] and renal failure [ROR = 8.66 (5.40, 13.90)] (Fig. 3). Clotrimazole had a strong positive signal for kidney damage in clinical applications. A pharmacological study found that long-term administration of large amounts of clotrimazole mainly manifested as damage to the adrenal cortex and liver^12^. This suggests that clotrimazole may cause nephrotoxicity in clinical practice.

For econazole, there was a large signal intensity for skin and subcutaneous tissue diseases, among which scarlatiniform rash had the strongest signal [ROR = 47 496.48 (14 946.01, 150 937.63)], followed by nail poisoning [ROR = 3423.23 (1062.81, 11 025.99)] (Fig. 3). Spontaneous abortion occurred most frequently (nine cases) [ROR = 19.62 (9.31, 41.34)], suggesting that skin and reproductive toxicity were the primary concerns with use of econazole.

For ketoconazole, the AEs with the highest frequency were chronic kidney disease [ROR = 11.64 (7.0, 19.37)], followed by alopecia [ROR = 7.88 (5.52, 11.31)] (Fig. 3). This suggests that ketoconazole causes a range of skin and subcutaneous tissue diseases, and nephrotoxicity is also a possibility.

Miconazole mainly caused diseases of the reproductive system and breast and diseases of the skin and subcutaneous tissue (Fig. 3). The most severe AE was vulvar edema [ROR = 1043.36 (636.18, 1803.50)], and the most frequent was vulvovaginal burning [ROR = 842.44 (579.19, 1276.23)]. Miconazole is particularly effective against *Candida*, and can be used for systemic treatment^16,30^.

### Comparing drug information leaflets

We sorted AEs signals of imidazoles according to ROR signal values by comparing drug information leaflets (Table 3) and analyzed positive signals (Supplementary Table 3).We found that cotrimoxazole causes neurological disease such as post-viral fatigue syndrome [2282.44 (10 236.93, 508.9)], and immune system diseases such as endocrine ophthalmopathy [464.68 (1114.93, 193.67)]. Econazole causes nail toxicity [3423.23 (11 025.99, 1062.81)] and adrenal insufficiency [166.33 (316.97, 87.28)]. Miconazole causes increased cortisol [625.29 (1388.52, 281.58)], hypothalamic pituitary adult axis suppression [1012.69 (2354.84, 435.5)], and antiphospholipid antibiotics [428.41 (1122.53, 163.5)]. Ketoconazole can lead to Jarisch–Herxheimer reaction [543.61 (1912.59, 154.51)].

**Table 3:** A positive signal not recorded in the drug information leaflets.

Clotrimazole	Adverse event	N	ROR
0–18	Tinea infection*	3	525.95 (1920.37, 144.04)
0–18	Granuloma*	4	236.64 (683.44, 81.94)
19–39	Laryngotracheal oedema*	4	2282.44 (10, 236.93, 508.9)
19–39	Post viral fatigue syndrome*	7	604.47 (1439.02, 253.91)
19–39	Pubic pain*	3	465.48 (1675.92, 129.29)
19–39	Eye infection toxoplasmal*	3	365.73 (1278.45, 104.63)
19–39	Hypervigilance*	6	224.59 (529.38, 95.28)
19–39	Plantar fasciitis*	6	210.84 (495.49, 89.72)
40–59	Angiodermatitis*	4	5087.07 (27, 849.93, 929.2)
40–59	Endocrine ophthalmopathy*	6	464.68 (1114.93, 193.67)
40–59	Toxic shock syndrome*	6	326.26 (767.28, 138.73)
40–59	Chemical burn of skin*	3	262.49 (865.03, 79.65)
40–59	Mean cell haemoglobin increased*	6	189.31 (436.25, 82.15)
≥ 60	Bladder transitional cell carcinoma*	8	291.66 (613.67, 138.62)

Econazole	Adverse event	N	ROR
19–39	Muscle necrosis*	4	2764.73 (8382.12, 911.91)
19–39	Myositis*	4	338.44 (961.27, 119.15)
40–59	Vulvovaginal candidiasis*	7	2261.48 (5305.21, 964.01)
≥ 60	Hypothalamic pituitary adrenal axis suppression*	8	4128.62 (9704.05, 1756.54)
≥ 60	Nail toxicity*	4	3423.23 (11, 025.99, 1062.81)
≥ 60	Adrenal insufficiency*	10	166.33 (316.97, 87.28)

Miconazole	Adverse event	N	ROR
19–39	Cortisol increased*	8	625.29 (1388.52, 281.58)
40–59	Endocrine ophthalmopathy*	6	446.35 (1070.71, 186.07)
40–59	Toxic shock syndrome*	6	313.39 (736.85, 133.29)
40–59	Cortisol increased*	3	261.21 (862.4, 79.12)
40–59	Mean cell haemoglobin increased*	6	181.84 (418.94, 78.92)
≥ 60	Xanthelasma*	5	1785.05 (5863.64, 543.42)
≥ 60	Hypothalamic pituitary adrenal axis suppression*	8	1012.69 (2354.84, 435.5)
≥ 60	Aspergillus test positive*	7	752.02 (1784.59, 316.9)
≥ 60	Antiphospholipid antibodies positive*	5	428.41 (1122.53, 163.5)

Ketoconazole	Adverse event	N	ROR
≥ 60	Jarisch–Herxheimer reaction*	3	543.61 (1912.59, 154.51)

## Discussion

Mucosal fungal infections are common clinically and can usually be adequately treated with azole compounds^22,23^. Azoles are a class of promising antifungal drugs that have been widely used in clinical practice^24^.

At present, there is no research indicating that exposure to imidazole drugs can lead to fetal developmental toxicity and renal toxicity. To investigate potential safety issues, we conducted a two-step analysis of the data. First, we screened and obtained 312 developmental toxicity keywords (Supplementary Table S2). And classify kidney and urinary system diseases based on MedDRA. Next, we conducted SOC distribution on the 9985 AE signals discovered, and found that they mainly involve 23 SOC. Our Renal and urinary disorders; pregnancy, purperium and perinal conditions signals. Were found in the SOC of all four drugs. Next, we analyzed the top 50 PTs from the top 10 SOCs. Then, we analyzed the top 50 PTs for each drug data ranking and segmented the data by age group: 0–18, 19–39, 40–59 and ≥ 60 years. We screened the Lowest Level Clotrimazole AEs included bladder transitional cell carcinoma [291.66 (613.67, 138.62)] and fetal death [10.28 (24.92, 4.24)]. Ketoconazole AEs included nephrogenic anemia [22.1 (59.36, 8.23)] and premature rupture of membranes [22.91 (46.45, 11.3). Miconazole AEs included hematuria [19.03 (30.48, 11.88)], neonatal sepsis [123.71 (274.76, 55.7)] and spontaneous abortion [5.98 (16.02, 2.24)]. Econazole AEs included acute kidney injury [4.41 (9.97, 1.95)] and spontaneous abortion [19.62 (41.34, 9.31)]. The imidazole drugs were analyzed by screening the top 50 PTs based on ROR intensity. Clotrimazole was associated with neurological disorders, such as laryngotracheal edema [2282.44 (10, 236.93, 508.9)], and immune system diseases, such as endocrine ophthalmopathy [464.68 (1114.93, 193.67)]. Econazole can cause nail toxicity [3423.23 (11, 025.99, 1062.81)] and adrenal insufficiency [166.33 (316.97, 87.28)]. Miconazole can cause increased cortisol [625.29 (1388.52, 281.58)], hypothalamic pituitary adrenal axis suppression [1012.69 (2354.84, 435.5)] and antiphospholipid antibodies [428.41 (1122.53, 163.5)]. Ketoconazole can cause Jarisch–Herxheimer reaction [543.61 (1912.59, 154.51)].

In comparison with our study, it was found that the frequency of adverse drug reactions was partially consistent with the adverse events observed for various drugs^25^. In this study, we conducted a retrospective study of azole drugs in the FAERS database, and detected the major AEs, with particular monitoring of their associated reproductive toxicity. Continued attention should be given to exploring how to use the clinical characteristics of AEs to provide early warning for post-marketing of drugs.

### Limitations

This study had several limitations that must be acknowledged. First, we have selectively selected the first four imidazole drugs that combine safety and efficacy, and may have missed the adverse reactions associated with other imidazole drugs. Second, FAERS is a passive and spontaneous AE database that receives voluntary reports from undefined populations^9,26^. Some studies claim that only 5% of serious AEs are actually submitted^27^^.^ Case reports are often incomplete, missing, or duplicated^12^. At the same time, some information that would have allowed more detailed analysis of each study is missing^28–31^. Finally, the AEs may have been related to the underlying disease being treated, or caused by some other drug being taken concurrently, or occurred for other reasons. The subjective judgment and interpretation of the reporters may also have an impact on the accuracy of the data. Therefore, when using FAERS data for causal relationship assessment, it is necessary to carefully review and verify the data.

## Conclusions

This study utilized the FAERS database to analyze potential AEs of representative drugs used for the treatment of VVC, and collected renal and developmental toxicity of imidazole drugs in clinical practice. These findings emphasize the importance of monitoring rare but severe AEs associated with imidazole drug use. At the same time, by comparing the instructions, it was found that AEs were not recorded in the instructions. Although the overall incidence of these events may be low, their clinical significance deserves careful consideration, especially in pregnant women or vulnerable populations with existing renal dysfunction. Healthcare providers must always be aware of the latest safety information about these drugs and adjust treatment decisions accordingly to minimize potential risks to patients. Long-term observation of these AEs is required to ensure the health and safety of patients treated with these drugs^32^.

### Supplementary Information

Supplementary Tables.

## Data Availability

This study utilized the FDA Adverse Event Reporting System (FAERS) database provided by the FDA. The dataset generated and analyzed during this study is available from the corresponding author upon reasonable request.
