# Supplementary material for: Possible adverse events of imidazole antifungal drugs during treatment of vulvovaginal candidiasis: analysis of the FDA Adverse Event Reporting System
Source: Sci Rep. 2024 Jun 24;14:14560. doi: 10.1038/s41598-024-63315-1 (PMC11196722; doi:10.1038/s41598-024-63315-1)
Supplement: Supplementary file 1 — Supplementary Tables. [file 41598_2024_63315_MOESM1_ESM.pdf]

Title: Possible adverse events of imidazole antifungal drugs during treatment of vulvovaginal candidiasis: Analysis of the FDA Adverse Event Reporting System

Authorship: Tianyu Zhou[a,b,c,d†], Chongze Chen[e†],Xiaowei Chen[f†], Bin Wang[a,b,c], Feng Sun[f], Wan Fang Li[d,g,h], Dong Liu[i*], and Hongtao Jin[d,g,h*]

a Department of Pharmacy , Shaanxi University of Chinese Medicine, Xianyang, China.

b Key Laboratory of Pharmacodynamics and Material Basis of Chinese Medicine of Shaanxi Administration of Traditional Chinese Medicine, Xianyang, China.

c Engineering Research Center of Brain Health Industry of Chinese Medicine, Universities of Shaanxi Province, Xianyang, China.

d New Drug Safety Evaluation Center, Institute of Materia Medica, Chinese Academy of Medical Sciences and Peking Union Medical College, Beijing, China.

e Department of Pharmacy,Fuzhou Changle People´s District Hospital,Fuzhou,Fujian,China.

f Department of Epidemiology and Biostatistics, School of Public Health, Peking University, Beijing, China.

g NMPA Key Laboratory for Safety Research and Evaluation of Innovative Drug, Beijing, China.

h Beijing Union Genius Pharmaceutical Technology Development Co. Ltd, Beijing, China.

i Center for drug evaluation, NMPA, Beijing, China.

*Authors to whom correspondence should be addressed.

†These authors contributed equally to this work.

Correspondence to: Hongtao Jin, New Drug Safety Evaluation Center, Institute of Materia Medica, Chinese Academy of Medical Sciences and Peking Union Medical College, Beijing, China.NMPA Key Laboratory for Safety Research and Evaluation of Innovative Drug, Beijing 102206, China.Beijing Union-Genius Pharmaceutical Technology Development Co. Ltd, Beijing, China. jinhongtao@imm.ac.cn

Supplementary Table 1 Signal detection algorithm

Method	Calculation formula	Conditions for inclusion
ROR	$ROR = \frac{a/c}{b/d}$<br>$95\%CI = e^{ln(ROR)\pm 1.96\sqrt{\frac{1}{a}+\frac{1}{b}+\frac{1}{c}+\frac{1}{d}}}$	a⩾3, 95%CI> 1
PRR	$PRR = \left[\frac{a}{a+b}\right] / \left[\frac{c}{c+d}\right]$<br>$95\%CI = e^{ln(PRR)\pm 1.96SE(lnPRR)}$<br>$\chi^2 = \frac{\left(\lvert ab - c\,\mathrm{d}\rvert - \frac{N}{2}\right)^2 \times N}{(a+b)(c+d)(a+c)(b+d)}$	a⩾3, PRR positive signal > 2, χ^2 > 4,95%CI> 1
BCPNN	$IC = log_2 \frac{a(a+b+c+\mathrm{d})}{(a+b)(a+c)}$<br>$\gamma = \gamma_{ij} \frac{(N+\alpha)(N+\beta)}{(a+b+\alpha_i)(a+c+\beta_j)}$<br>$E(IC) = log_2 \frac{(a+\gamma_{ij})(N+\alpha)(N+\beta)}{(N+\gamma)(a+b+\alpha_i)(a+C+\beta_j)}$<br>$V(IC) = \left(\frac{1}{\ln 2}\right)^2 \left(\frac{N-\alpha+\gamma-\gamma_{ij}}{(\alpha+\gamma_{ij})(1+N+\gamma)} + \frac{N-a-b+\alpha-\alpha_i}{(a+b+\alpha_i)(1+N+\alpha)} + \frac{N-a-C+\beta-\beta_j}{(a+C+\beta_j)(1+N+\beta)}\right)$<br>$SD = \sqrt{V(IC)}$<br>$IC025 = \mathrm{E(IC)} - 2\mathrm{SD}$	IC025 > 0

a is the number of target AEs reports for the target drug; b is the number of other AE reports of the target drug; c is the number of target AE reports of other drugs; d is the number of other AE reports of other drugs; $N = a + b + c + d$, γ, γ_{ij} is the parameter of Dicichlet distribution; α_i, α, β_j,β is the parameter of Beta distribution; IC025 is the lower limit of 95% CI of IC, Hypothesis $\alpha = \beta = 2$, $\gamma_{ij} = \beta_j = \alpha_i = 1$.

Supplementary Table 2 Keywords for reproductive toxicity

Keywords for reproductive toxicity
Autoinflammation with infantile enterocolitis; Abortion spontaneous incomplete; Spontaneous rupture of membranes; Abortion spontaneous complete; Abortion spontaneous; Abortion spontaneous complete complicated; Abortion spontaneous complicated; Missed labour; Bronchopulmonary dysplasia; Sinusoidal foetal heart rate pattern; Zika virus associated ocular birth defect; Zika virus associated birth defect; Abortion early; Preterm premature rupture of membranes; Premature baby death; Retinopathy of prematurity; Premature baby; Premature labour; Primary familial hypomagnesaemia; Infantile vomiting; Infantile back arching; Maternal drugs affecting foetus; Infantile acropustulosis; Infant sedation; Neuroendocrine cell hyperplasia of infancy; Subaponeurotic cerebrospinal fluid collection of infancy; Infant dyschezia; Infantile spitting up; Infantile apnoea; Infantile colic; Transient hypogammaglobulinaemia of infancy; Sudden infant death syndrome; Amniorrhoea; Oligohydramnios; Polyhydramnios; Meconium in amniotic fluid; Amniotic cavity disorder; Amniotic cavity infection; Amniotic band syndrome; Prolonged rupture of membranes; Fontanelle bulging; Fontanelle depressed; Neonatal gastrointestinal disorder; Neonatal gastrointestinal haemorrhage; Neonatal alloimmune thrombocytopenia; Periventricular haemorrhage neonatal; Neonatal testicular torsion; Neonatal intestinal perforation; Cyanosis neonatal; Purpura neonatal; Subarachnoid haemorrhage neonatal; Myasthenia gravis neonatal; Neutropenia neonatal; Erythema toxicum neonatorum; Neonatal asphyxia; Neonatal oversedation; Tremor neonatal; Transient neonatal pustular melanosis; Dyskinesia neonatal; Hyperkinesia neonatal; Hypokinesia neonatal; Subdural haemorrhage neonatal; Neonatal complications of substance abuse; Drug withdrawal syndrome neonatal; Ophthalmia neonatorum; Circulatory failure neonatal; Blood incompatibility haemolytic anaemia of newborn; Thrombocytopenia neonatal; Thrombophlebitis neonatal; Neonatal haemochromatosis; Lymphocytopenia neonatal; Neonatal behavioural syndrome; Cardiac arrest neonatal; Cardio-respiratory arrest neonatal; Neonatal cardiac failure; Arrhythmia neonatal; Cardiomyopathy neonatal; Neonatal tachycardia; Bradycardia neonatal; Neonatal bacterial pneumonia; Neonatal aspiration; Neonatal anuria; Late metabolic acidosis of newborn; Peripheral oedema neonatal; Cephalhaematoma; Newborn head moulding; Neonatal hypoacusis; Weight decrease neonatal; Poor weight gain neonatal; Hypothermia neonatal; Disturbance of thermoregulation of newborn; Neonatal diabetes mellitus; Death neonatal; Oedema neonatal; Neonatal tetany; Somnolence neonatal; Vision abnormal neonatal; Blood loss anaemia neonatal; Renal impairment neonatal; Renal failure neonatal; Adrenocortical insufficiency neonatal; Adrenal insufficiency neonatal; Breast engorgement in newborn; Haemolysis neonatal; Hypoxic ischaemic encephalopathy neonatal; Neonatal anoxia; Neonatal pneumothorax; Tetanus neonatorum; Anaemia neonatal; Rash neonatal; Neonatal mucocutaneous herpes simplex; Sepsis neonatal; Coagulation disorder neonatal; Urinary tract infection neonatal; Neonatal candida infection; Intraventricular haemorrhage neonatal; Meningitis neonatal; Encephalopathy neonatal; Disseminated intravascular coagulation in newborn; Anaesthetic complication neonatal; Intracranial haemorrhage neonatal; Conjunctivitis gonococcal neonatal; Granulocytopenia neonatal; Neonatal tachyarrhythmia; Neonatal Crohn's disease; Coxsackie viral disease of the newborn; Neonatal bacteraemia; Venous thrombosis neonatal; Pseudomenstruation neonatal; Neonatal thyrotoxicosis; Neonatal hypoparathyroidism; Neonatal disorder; Agitation neonatal; Neonatal deformity; Hypertonia neonatal; Hypotonia neonatal; Coma neonatal; Jaundice neonatal; Neonatal bradyarrhythmia; Necrotising enterocolitis neonatal; Gangrene neonatal; Neonatal respiratory depression; Neonatal respiratory acidosis; Neonatal respiratory alkalosis; Respiratory disorder neonatal; Neonatal respiratory arrest; Neonatal respiratory failure; Neonatal dyspnoea; Neonatal respiratory distress syndrome; Neonatal respiratory distress; Neonatal

tachypnoea; Respiratory tract haemorrhage neonatal; Polycythaemia neonatorum; Neonatal lupus erythematosus; Melaena neonatal; Administration site reaction neonatal; Neonatal hyperglycaemia; Hyperbilirubinaemia neonatal; Neonatal infective mastitis; Diarrhoea infectious neonatal; Neonatal infection; Neonatal hepatomegaly; Hepatitis neonatal; Hepatocellular damage neonatal; Hepatosplenomegaly neonatal; Diarrhoea neonatal; Neonatal pneumonia; Hypoventilation neonatal; Pulmonary oedema neonatal; Neonatal alveolar aeration excessive; Pulmonary haemorrhage neonatal; Atelectasis neonatal; Neonatal hypoxia; Fever neonatal; Neonatal deafness; Neonatal multi-organ failure; Transient tachypnoea of the newborn; Neonatal toxicity; Neonatal sinus tachycardia; Neonatal sinus bradycardia; Neonatal epileptic seizure; Neonatal seizure; Neonatal hypotension; Hypoglycaemia neonatal; Neonatal hyponatraemia; Neonatal hypocalcaemia; Neonatal cholestasis; Meningoencephalitis herpes simplex neonatal; Herpes simplex virus conjunctivitis neonatal; Cerebral haemorrhage neonatal; Haemorrhagic disease of newborn; Haemorrhage neonatal; Newborn persistent pulmonary hypertension; Neonatal intestinal dilatation; Neonatal intestinal obstruction; Disseminated neonatal herpes simplex; Constipation neonatal; Inclusion conjunctivitis neonatal; Neonatal leukaemia; Leukopenia neonatal; Rhesus haemolytic disease of newborn; Group B streptococcus neonatal sepsis; ABO haemolytic disease of newborn; Oblique presentation; Small for dates baby; Small size placenta; Hypertension neonatal; Confined placental mosaicism; Pre-eclampsia; Threatened uterine rupture; Abortion threatened; Habitual abortion; Suck-swallow breathing coordination disturbance; Feeding intolerance; Risk of future pregnancy miscarriage; Perinatal brain damage; Abortion late; Abortion complete; Campomelic syndrome; Breech presentation; Breech delivery; Asymmetric gluteal fold; Scalp haematoma; Asynclitic presentation; Cephalo-pelvic disproportion; Alloimmunisation; Isoimmune haemolytic disease; Diabetic foetopathy; Foetal heart rate disorder; Foetal heart rate deceleration abnormality; Foetal heart rate acceleration abnormality; Baseline foetal heart rate variability disorder; Foetal malpresentation; Foetal malposition; Unstable foetal lie; Metastases to placenta; Placental neoplasm; Premature separation of placenta; Placental polyp; Placental transfusion syndrome; Placental chorioangioma; Placental cyst; Placental disorder; Retained placenta or membranes; Placental necrosis; Placental lake; Retroplacental haematoma; Placental insufficiency; Placental infarction; Placental calcification; Placental hypertrophy; Placental dysplasia; Malignant neoplasm of placenta; Premature rupture of membranes; Meconium increased; Meconium abnormal; Meconium peritonitis; Meconium ileus; Meconium aspiration syndrome; Meconium plug syndrome; Meconium cyst; Foetal hypokinesia; Foetal malnutrition; Foetal tobacco syndrome; Persistent foetal circulation; Angiotensin converting enzyme inhibitor foetopathy; Foetal vascular malperfusion; Foetal cardiac arrest; Foetal cardiac disorder; Foetal arrhythmia; Tachycardia foetal; Bradycardia foetal; Enlarged foetal cisterna magna; Foetal damage; Foetal acidosis; Foetal death; Hydrops foetalis; Foetal growth abnormality; Foetal growth restriction; Delayed foetal renal development; Foetal renal impairment; Foetal chromosome abnormality; Foetal compartment fluid collection; Foetal anaemia; Harlequin foetus; Foetal cerebrovascular disorder; Cerebral infarction foetal; Cerebral haemorrhage foetal; Foetal dystocia; Virilism foetal; Anaesthetic complication foetal; Foetal retinoid syndrome; Foetal tachyarrhythmia; Foetal anticonvulsant syndrome; Foetal alcohol syndrome; Foetal alcohol spectrum disorder; Foetal distress syndrome; Foetal disorder; Foetal malformation; Foetal warfarin syndrome; Radiation injury affecting foetus; Hypopituitarism foetal; Haemorrhage foetal; Erythroblastosis foetalis; Foetal arm prolapse; Foetal methotrexate syndrome; Foetal-maternal haemorrhage; Foetal movement disorder; Increased foetal movements; Meconium stain; Stillbirth; Poor sucking reflex

Supplementary Table 3 Drug information leaflets for imidazole drug AEs

Clotrimazole	Econazole	Miconazole	Ketoconazole
Paresthesia;Rash;Edema; Secondary infection; Itching; Irritation; Dryness; Folliculitis; Hypertrichosis; Acneiform eruptions; Hypopigmentation; Perioral dermatitis; Allergic contact dermatitis; Maceration of the skin; Secondary infection; Skin atrophy; Striae; Miliaria; Growth retardation; Benign intracranial hypertension; Local cutaneous reactions; Hyperglycemia; Erythema; Blistering; Peeling; Edema; Pruritus; General irritation of the skin; Lower abdominal; Back or shoulder pain; Fever; Chills; Nausea; Vomiting; Foul-smelling vaginal discharge	Burning; Itching; Stinging; Erythema; Pruritic rash	Allergic reactions; Vaginal secretions; Local stimulation; Itching and burning sensation; Pelvic spasm; Urticaria; Skin papules; Angioneurotic edema; Eczema; Vaginal stimulation; Discomfort at the administration site	Allergic reactions; Rash; Nausea; Burn of external genitalia; Vaginal dryness; Skin redness and swelling; Blisters; Spalling; Wheezing; A tight throat; Difficulty breathing or speaking; Swelling of the tongue or throat; Abnormal hoarseness in voice; Lips; Oral cavity

Supplementary Table 4 AEs results of imidazoles were collected by comparison with drug information leaflets

Clotrimazole	Adverse event	N	ROR	PRR	χ2	IC025
0-18	tinea infection*	3	525.95(1920.37,144.04)	507.2(1785.54,144.07)	14639.41	7.09
0-18	granuloma*	4	236.64(683.44,81.94)	225.42(622.27,81.66)	53072.38	6.16
0-18	vulvovaginal burning sensation	3	165.27(548.21,49.83)	159.41(508.26,49.99)	58232.55	5.8
19-39	laryngotracheal oedema*	4	2282.44(10236.93,508.9)	2255.83(10041.7,506.77)	1507.423	8.08
19-39	post viral fatigue syndrome*	7	604.47(1439.02,253.91)	592.16(1391.28,252.03)	23816.03	7.15
19-39	pubic pain*	3	465.48(1675.92,129.29)	461.42(1646.71,129.29)	13318.09	6.98
19-39	eye infection toxoplasmal*	3	365.73(1278.45,104.63)	362.54(1255.91,104.66)	18132	6.75
19-39	hypervigilance*	6	224.59(529.38,95.28)	220.68(513.3,94.88)	67341.8	6.08
19-39	plantar fasciitis*	6	210.84(495.49,89.72)	207.17(480.41,89.34)	72341.63	6
40-59	Angiodermatitis*	4	5087.07(27849.93,929.2)	5038.63(27435.73,925.35)	942.5138	8.81
40-59	endocrine ophthalmopathy*	6	464.68(1114.93,193.67)	458.06(1087.45,192.94)	68218.93	7.05
40-59	toxic shock syndrome*	6	326.26(767.28,138.73)	321.61(748.19,138.25)	102765.6	6.63
40-59	chemical burn of skin*	3	262.49(865.03,79.65)	260.62(852.27,79.7)	63939.45	6.51
40-59	mean cell haemoglobin increased*	6	189.31(436.25,82.15)	186.62(425.3,81.88)	187627.4	5.94
⩾60	bladder transitional cell carcinoma*	8	291.66(613.67,138.62)	288.24(601.82,138.05)	100842.3	6.42
Econazole	Adverse event	N	ROR	PRR	χ2	IC025
19-39	muscle necrosis*	4	2764.73(8382.12,911.91)	2481.27(6818.84,902.9)	293614.1	9.48
19-39	myositis*	4	338.44(961.27,119.15)	303.83(776.92,118.82)	2844088	6.76
40-59	rash scarlatiniform	7	47496.48(150937.63,14946.01)	37421.68(105418.58,13284.02)	74632.65	12.22
40-59	vulvovaginal candidiasis*	7	2261.48(5305.21,964.01)	1781.98(3518.64,902.47)	3798060	9.1
⩾60	hypothalamic pituitary adrenal axis suppression*	8	4128.62(9704.05,1756.54)	3930.89(8983.67,1720)	90954.43	9.72
⩾60	nail toxicity*	4	3423.23(11025.99,1062.81)	3341.26(10548,1058.4)	53841.73	9.6
⩾60	adrenal insufficiency*	10	166.33(316.97,87.28)	156.43(287.01,85.26)	4368726	5.7

Miconazole	Adverse event	N	ROR	PRR	χ^2	IC025
19-39	cortisol increased*	8	625.29(1388.52,281.58)	607.64(1325.98,278.46)	41133.65	7.26
40-59	endocrine ophthalmopathy*	6	446.35(1070.71,186.07)	440.23(1045.35,185.4)	65563.63	6.99
40-59	toxic shock syndrome*	6	313.39(736.85,133.29)	309.1(719.23,132.84)	98765.78	6.57
40-59	cortisol increased*	3	261.21(862.4,79.12)	259.42(850.18,79.16)	59059.65	6.49
40-59	mean cell haemoglobin increased*	6	181.84(418.94,78.92)	179.35(408.83,78.68)	180325	5.89
⩾60	xanthelasma*	5	1785.05(5863.64,543.42)	1771.45(5790.2,541.96)	5840.322	8.19
⩾60	hypothalamic pituitary adrenal axis suppression*	8	1012.69(2354.84,435.5)	1000.35(2309.89,433.22)	23138.08	7.76
⩾60	aspergillus test positive*	7	752.02(1784.59,316.9)	744.01(1753.53,315.68)	29926.51	7.49
⩾60	antiphospholipid antibodies positive*	5	428.41(1122.53,163.5)	425.15(1107.19,163.25)	42523	6.93
⩾60	bronchopulmonary aspergillosis allergic*	7	217.97(476.1,99.79)	215.66(467.49,99.48)	131224.3	6.08
Ketoconazole	Adverse event	N	ROR	PRR	χ^2	IC025
0-18	vulval oedema	15	3603.48(10059.8,1290.79)	3230.81(8773.08,1189.79)	1084.193	7.88
0-18	vulvovaginal burning sensation	19	1239.18(2396.28,640.82)	1076.94(1991.38,582.41)	9682.903	7.36
0-18	vaginal swelling	4	402.7(1279.76,126.71)	391.61(1215.64,126.16)	7908.941	6.56
0-18	vaginal burning sensation	4	340.74(1057.74,109.76)	331.37(1004.25,109.34)	9891.235	6.4
0-18	vulvovaginal swelling	3	274.9(984.61,76.75)	269.23(944.1,76.78)	9489.12	6.23
0-18	oedema genital	3	206.17(715.35,59.42)	201.93(685.56,59.48)	13610.76	5.94
0-18	vulvovaginal discomfort	6	192.54(465.07,79.72)	184.62(432.2,78.86)	31247.5	5.74
0-18	vulvovaginal pruritus	5	185.86(485.99,71.08)	179.49(456.13,70.63)	26763.85	5.73
19-39	vulval oedema	130	5647.32(9534.18,3345.04)	4885.89(8180.66,2918.09)	965.8802	7.38
19-39	vaginal burning sensation	95	844.86(1151.88,619.67)	761.7(1024.17,566.49)	19558.07	6.72
19-39	vulvovaginal burning sensation	162	812.96(1028.24,642.75)	676.51(839.12,545.41)	40341.49	6.64
19-39	vaginal exfoliation	4	603.84(2417.98,150.8)	601.34(2401.02,150.61)	916.8816	6.46

19-39	vaginal swelling	64	515.21(720.34,368.5)	481.07(664.2,348.43)	26325.92	6.39
19-39	vaginal erythema	14	502.5(1022.33,247)	495.22(1001.8,244.8)	5265.88	6.4
19-39	vulvovaginal hypoaesthesia	3	452.41(2024.14,101.12)	451.01(2012.47,101.07)	1048.854	6.28
19-39	vulvovaginal erythema	21	403.4(702.08,231.78)	394.63(681.81,228.41)	11232.88	6.22
19-39	vulvovaginal swelling	71	362.83(488.68,269.39)	336.18(446.37,253.2)	48510.53	6.09
19-39	genital pruritus female	35	346.63(526.6,228.16)	334.08(502.56,222.08)	23924.39	6.08
19-39	vaginal pain	28	321.1(509.12,202.52)	311.81(490.01,198.41)	20952.98	6.02
19-39	oedema genital	11	223.02(446.33,111.44)	220.49(438.7,110.82)	12747.01	5.7
19-39	genital swelling	6	201.7(509.22,79.89)	200.45(503.88,79.74)	7663.757	5.63
19-39	vulvovaginal inflammation	6	201.7(509.22,79.89)	200.45(503.88,79.74)	7663.757	5.63
19-39	vulvovaginal discomfort	93	195.77(249.01,153.91)	176.98(221.06,141.68)	146163.9	5.43
19-39	genital burning sensation	9	195.1(414.56,91.82)	193.29(408.52,91.45)	12276.49	5.57
19-39	vulvovaginal pruritus	76	191.47(249.33,147.04)	176.46(226.07,137.73)	119802	5.43
19-39	vulvovaginal pain	71	154.58(201.9,118.36)	143.27(184.15,111.47)	144070.3	5.2
40-59	vulval oedema	33	4233.64(7951.88,2254.02)	4009.08(7453.98,2156.26)	6577.086	8.51
40-59	vaginal burning sensation	49	1924.74(2895.79,1279.32)	1773.2(2625.2,1197.71)	38264.14	8.08
40-59	vaginal swelling	22	1491.89(2647.02,840.84)	1439.16(2525.32,820.16)	23017.61	7.92
40-59	vulvovaginal swelling	34	774.27(1166.75,513.81)	732(1085.76,493.5)	92701.74	7.34
40-59	vulvovaginal burning sensation	96	714.92(916.23,557.84)	604.74(752.97,485.68)	337340.1	7.13
40-59	vaginal erythema	5	659.45(1855.39,234.39)	654.16(1829.46,233.91)	14756.35	7.27
40-59	genital pruritus female	17	560.85(974.13,322.9)	545.55(936.75,317.72)	66941.89	7.05
40-59	vaginal mucosal blistering	7	523.52(1224.55,223.82)	517.64(1201.93,222.93)	28689.74	7.03
40-59	vaginal pain	9	501.02(1056.8,237.53)	493.79(1032.84,236.07)	39539.54	6.97
40-59	oedema genital	8	430.73(938.49,197.69)	425.21(919.04,196.73)	42176.74	6.81
40-59	vulvovaginal erythema	11	352.68(677.74,183.53)	346.46(659.42,182.03)	74873.22	6.56
40-59	vulvovaginal pain	44	306.13(425.61,220.2)	284.55(387.73,208.82)	381599.4	6.28

40-59	vulvovaginal discomfort	46	271.58(374.08,197.16)	251.57(339.4,186.46)	459127.3	6.12
40-59	chemical burn	4	195.63(552.09,69.32)	194.38(545.28,69.29)	51898.23	6
40-59	vulvovaginal pruritus	33	188.71(272.56,130.65)	178.75(253.64,125.97)	481544.4	5.7
⩾60	vulval oedema	8	3167.61(9157.63,1095.67)	3125.04(8979.21,1087.61)	5057.646	8.63
⩾60	vaginal burning sensation	12	1063.1(2108.61,535.98)	1041.68(2046.39,530.25)	42185.89	7.84
⩾60	vulvovaginal burning sensation	96	602.71(764.19,475.35)	505.62(620.92,411.74)	855630.2	7.04
⩾60	jarisch-herxheimer reaction*	3	543.61(1912.59,154.51)	540.87(1893.18,154.52)	22882.83	7.29
⩾60	vaginal erosion	4	496.76(1464.62,168.49)	493.43(1446.08,168.37)	34868.84	7.16
⩾60	vaginal mucosal blistering	3	441.68(1519.83,128.36)	439.46(1504.27,128.38)	29630.47	7.08
⩾60	vulvovaginal injury	6	417.81(998.93,174.75)	413.61(981.51,174.29)	65745.47	6.91
⩾60	vulvovaginal swelling	15	414.52(721.34,238.21)	404.1(694.85,235.01)	171894.1	6.81
⩾60	vulvovaginal pain	34	254.51(365.71,177.12)	240.03(338.46,170.22)	703514.3	6.13
⩾60	vulvovaginal rash	3	164.34(531.2,50.84)	163.52(525.62,50.87)	92022.36	5.92

* Adverse reactions not recorded in drug information leaflets.
